# Enamel Polish

**Published:** 1897-01

**Authors:** 


### Enamel Polish.

We have, for some time, been using a preparation which the maker prefers to call Enamel Restorer. It is a preparation which gives a very fine polish to enamel and dentine and porcelain teeth as well, wherever the surface may have been ground for any purpose with ordinary corundum wheels or dressed with files, etc. It also gives a very beautiful surface to enamel even where it has not been touched. We have found it the best material for this purpose that we have ever used, and are sure that every one, both patient and operator, will be delighted with it. It is best used with moose hide or leather disks. It is obtainable at the dental depots.

